# High Expression Achievement of Active and Robust Anti-β2 microglobulin Nanobodies via *E.coli* Hosts Selection

**DOI:** 10.3390/molecules24162860

**Published:** 2019-08-07

**Authors:** Da Li, Fangling Ji, Chundong Huang, Lingyun Jia

**Affiliations:** Liaoning Key Laboratory of Molecular Recognition and imaging, School of Bioengineering, Dalian University of Technology, No.2 Linggong Road, Dalian 116023, China

**Keywords:** anti-β2MG VHHs, *E. coli* expression, Rosetta-gami B (DE3) pLysS

## Abstract

Nanobodies (VHHs) overcome many of the drawbacks of conventional antibodies, and the related technologies represent state-of-the-art and advanced applications in scientific research, pharmaceuticals, and therapies. In terms of productivity and economic cost, the cytoplasmic expression of VHHs in *Escherichia coli* (*E. coli*) is a good process for their recombinant production. The cytoplasmic environment of the host is critical to the affinity and stability of the recombinant VHHs in soluble form, yet the effects have not been studied. For this purpose, recombinant anti-β2 microglobulin VHHs were constructed and expressed in four commercialized *E. coli* hosts, including BL21 (DE3), Rosetta-gami B (DE3) pLysS, Origami 2 (DE3) and SHuffle T7 Express. The results showed that anti-β2 microglobulin (β2MG) VHHs expressed in different hosts exhibited distinctive differences in the affinity and structural characteristics. The VHHs expressed in Rosetta-gami B (DE3) pLysS possessed not only the greatest affinity of (equilibrium dissociation constant) *K_D_* = 4.68 × 10^−8^ M but also the highest yields compared with the VHHs expressed in BL21 (DE3), Origami 2 (DE3) and SHuffle T7 Express. In addition, the VHHs expressed in Rosetta-gami B (DE3) pLysS were more stable than the VHHs expressed in the rest three hosts. Thus far, we have successfully realized the high expression of the active and robust anti-β2MG VHHs in Rosetta-gami B (DE3) pLysS. The underlying principle of our study is able to guide the expression strategies of nanobodies on the context of industrial large-scale production.

## 1. Introduction

Nanobodies, different from the conventional antibody, are a distinct format of antibody fragments. They are derived from heavy-chain-only antibodies (HCAbs) which naturally occur in sera of Camelidae. The antigen-binding fragments of HCAbs are comprised in the variable domains of the heavy chain (VHHs), with a molecular size of only 15 kDa, and are also known as nanobodies [1]. Nanobodies are the smallest, intact, functional antigen-binding fragments and have been used widely in different applications [2,3,4,5]. VHHs are generally well-expressed at a low cost in prokaryotic systems. Since *E. coli* is by far the most popular host for the biopharmaceutical production of heterologous recombinant proteins, for low cost and Food and Drug Administration (FDA) approved status for human applications [6], most VHHs have been periplasmically or cytoplasmically produced in *E. coli* [7]. Generally, cytoplasmic VHH production yields are higher [8,9,10,11,12]. Though periplasmic extracts are preferred by Muyldermans [13] because their oxidizing environment forms disulfide bonds properly and their purification is straightforward, we hope our anti-β2 microglobulin (β2MG) VHH would be robust enough to enable large-scale production in the soluble and functional form in the cytoplasm of *E. coli*.

In recent years, commercialized *E. coli* host cells including the BL21 (DE3) strain [14,15,16], the Rosetta-gami B (DE3) pLysS strain [17,18], the Origami 2 (DE3) strain [19] and SHuffle T7 Express [20,21] have been frequently used in VHHs’ expression. These cells are genetically engineered to facilitate the production of the heterogenous protein. However, there are still many differences in the intracellular environment of these *E. coli* hosts, leading to the variations in the yield, structure and properties of the cytoplasmic heterogenous proteins [22]. Nevertheless, the exploration of the expression of VHHs almost focuses on the yield rather than the affinity, stability, and structural conformation.

β2 microglobulin (β2MG) has been identified as the major composition of the insoluble amyloid fibrils that causes dialysis-related amyloidosis (DRA) [23]. As the anti-β2MG nanobody binds with β2MG with remarkable high affinity, it becomes a highly valued protein for multiple applications in DRA, including fundamental research, diagnosis, prevention, and therapy. The anti-β2MG VHH (CNb1; the sequence information of which can be found in the Supplementary Materials) we used was generated in our lab, and it possessed a better solvability, stability, and affinity than the other anti-β2MG VHHs [24,25] we had selected from the phage display libraries before. It could become an extremely important tool in the treatment of DRA, such as a β2MG-adsorbent material and a β2MG diagnostic kit.

Here, the paper aims at producing high affinity and robust anti-β2MG VHHs for potential industrial production. We thus constructed the anti-β2MG VHH gene onto the pET23a vector and expressed the VHHs in four different *E. coli* hosts, including BL21 (DE3), Rosetta-gami B (DE3) pLysS, Origami 2 (DE3) and SHuffle T7 Express. The affinities of different anti-β2MG VHHs were determined by surface plasmon resonance (SPR), as were thermal and chemical stability by circular dichroism (CD) and protease resistance via trypsin digestion after we characterized their secondary structures. Based on these results, we clearly observed diverse biochemical properties among different cytoplasmic expressed anti-β2MG VHHs, although all VHHs were in soluble forms. As a result, our study enables the selection of the cytoplasmic expression of VHHs in *E. coli* intention for potential large-scale production.

## 2. Results

### 2.1. Expression of the Anti-β2MG VHHs in Four E. coli Hosts

**Figure 1:**
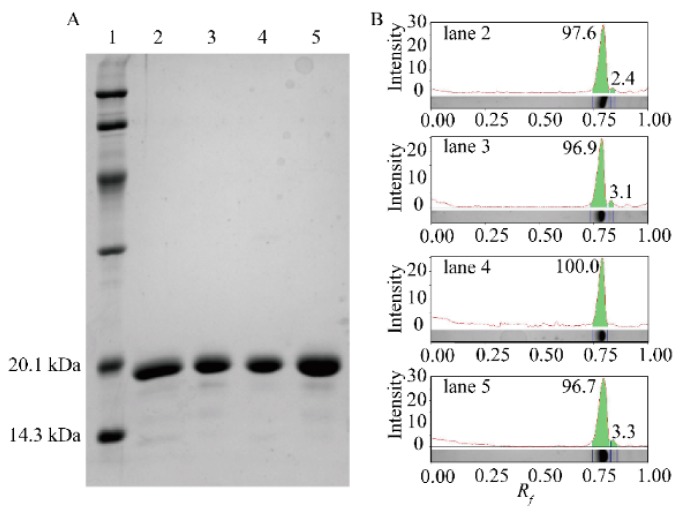
Purification of nanobodies (VHHs.) (**A**) SDS-PAGE analysis of purified VHH. Lane 1, protein ladder. Lanes 2–5, size-exclusion chromatography (SEC) elution fractions of *E. coli* BL21 (DE3), SHuffle T7 Express, Origami 2(DE3) and Rosetta-gami B (DE3) pLysS. (**B**) Quantitative analysis of SDS-PAGE using Imagelab software. The purity of *E. coli* BL21 (DE3), SHuffle T7 Express, Origami 2 (DE3) and Rosetta-gami B (DE3) pLysS was sequentially displayed from top to bottom.

As shown in Figure 1, the successful expression and purification of recombinant VHHs with a molecular weight of about 16 kDa was achieved. The result of an Image Lab^TM^ software analysis revealed that the purity of VHHs was above 95%. The final yield of soluble VHHs per liter culture when reached identical OD_600 nm_ were 4.8–5.2 mg (BL21 (DE3)), 8.2–9.5 mg (Origami 2 (DE3)), 47.5–52.5 mg (SHuffle T7 Express), and 54.5–57.0 mg (Rosetta-gami B (DE3) pLysS), respectively. It is obvious that much higher yield of VHHs were produced by SHuffle T7 Express and Rosetta-gami B (DE3) pLysS, more than ten times of that of BL21 (DE3).

The difference in the yield of soluble VHHs in four hosts might relate to their original sources. The Origami 2 (DE3) strain had a lower yield (9 mg/L) because it is an *E. coli* K-12 derivative. The other strains are *E. coli* B strain derivatives. As the *E. coli* B strain is deficient in the ion protease and has a lack of the ompT outer membrane protease which can degrade proteins during purification, the SHuffle T7 Express strain and the Rosetta-gami B (DE3) pLysS strain exhibited a higher level of expression than Origami 2 (DE3). Furthermore, the rare codons provided by the pLysS plasmid of Rosetta-gami B (DE3) pLysS improved production while avoiding the appearance of inclusion bodies caused by excessive expression. Thus, the Rosetta-gami B (DE3) pLysS strain had the highest soluble yield of these hosts. Though BL21 (DE3) is an *E. coli* B strain derivative and has a high yield of VHH, its cytoplasm has a less oxidizing environment, leading to VHHs predominantly present in the inclusion bodies and a a low soluble yield.

### 2.2. Affinity of VHHs

Affinity is the most important property of activity of the VHHs, so we evaluated the affinity of VHHs binding toward human β2MG using the SPR technique. As shown in Table 1, the (equilibrium dissociation constant) *K_D_* value of VHH expressed by Rosetta-gami B (DE3) pLysS was determined to be 4.60 × 10^−8^ M, two times greater than that of the VHHs expressed in BL21 (DE3) and 1.52 times and 1.46 times better than the VHHs expressed in SHuffle T7 Express and the VHHs expressed in Origami 2 (DE3), respectively.

**Figure 2:**
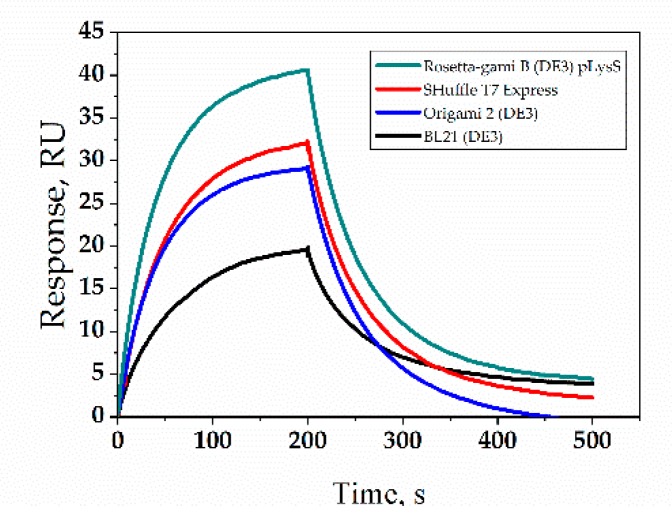
Binding kinetics of anti-β2 microglobulin (β2MG) VHHs expressed in different hosts. The protein concentration was the same at 0.5 μg/mL.

Considering their kinetics characterized (Figure 2 and Table 1), the dissociation rates of the VHHs expressed in SHuffle T7 Express and Origami 2 (DE3) were matched with Rosetta-gami B (DE3) pLysS, while the latter displayed a more rapid association rate, meaning that the VHHs expressed in Rosetta-gami B (DE3) pLysS bound antigens faster. The VHHs expressed in BL21 (DE3) displayed slower association and dissociation rates, resulting in a lower *K_D_*.

### 2.3. Secondary Structure Analysis for the VHHs

**Figure 3:**
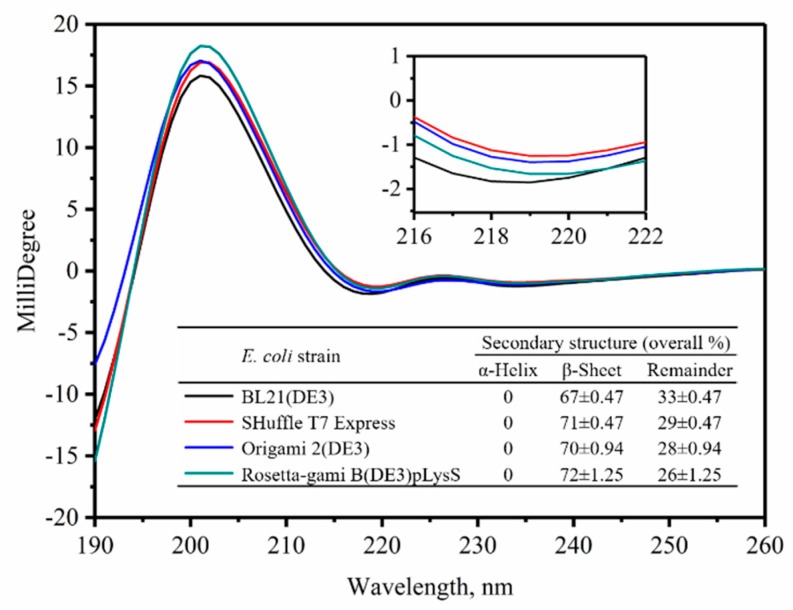
Far-UV spectra of VHHs expressed by different *E. coli* hosts. The inset was the scope of 216–222 nm. Secondary structures of VHHs were analyzed in Dicroprot, fitted by Contin (Linear Combination).

The affinity function of a protein is determined by the structure of the protein. As we found out, the affinities were different among the four type VHHs (Figure 2 and Table 1); there should be clues from the structures of the VHHs to explain the differences. In order to compare the secondary structure of VHHs expressed in four host cells, CD measurements were performed. The CD spectrum showing the ellipticity of α-helix and β-sheet was obtained at the wavelength of 190–260 nm (Figure 3). The fractions of the various secondary-structure elements of the four type VHHs derived from the CD spectra are summarized in the Figure 3 inset. The fitting was performed by using the raw data. An analysis of the CD spectra revealed that the β-sheet is the most dominant secondary-structure element of all four VHHs. The β-sheet of the VHHs expressed in BL21 (DE3) (67%) was less than that of the VHHs expressed in Rosetta-gami B (DE3) pLysS (72%). The change in the content of β-sheet was possibly due to the incomplete formation of disulfide bonds caused by the oxidizing environment of BL21 (DE3) [22].

### 2.4. Resistance to Trypsin Digestion

**Figure 4:**
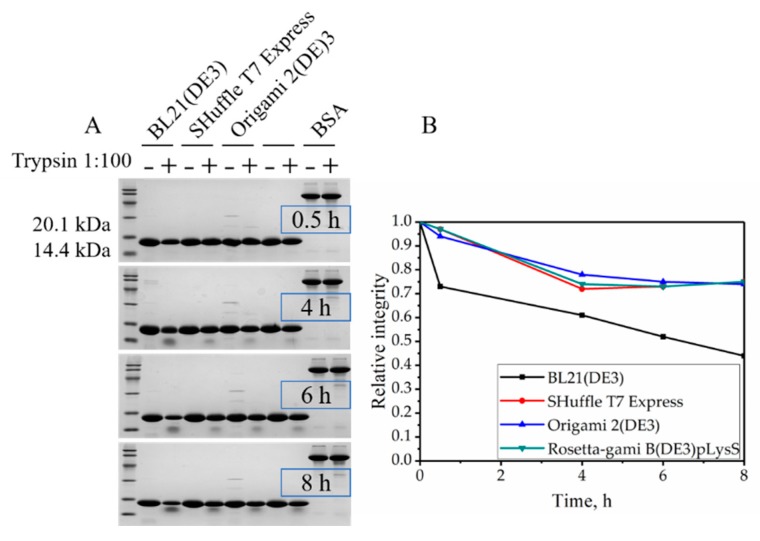
Digestion of VHHs with trypsin. (**A**) At various time points, aliquots (20 μg of protein) were subject to SDS-PAGE. BSA as control. (**B**) Degradation rate of VHHs with trypsin at various digestion time points.

Proteins with their natural structures are normally resistant to trypsin digestion. Thus, we tested these four VHHs with trypsin digestion. As is shown in Figure 4, the VHH expressed by BL21 (DE3) possessed relatively poor protease resistance after incubation, only retaining 73% of its integrity. However, the VHHs expressed by Rosetta-gami B (DE3) pLysS, Origami 2 (DE3), and SHuffle T7 Express retained 97%, 94% and 97% of their integrity, respectively, indicating the expression environment of BL21 (DE3) might not be suitable for the VHH to fold in a natural form. The percentages of integrity were calculated by gray scanning of the band densities in Figure 4. This is consistent with the CD measurement of secondary structure. Moreover, further degradation after 4 h of the VHHs expressed by Rosetta-gami B (DE3) pLysS, Origami 2 (DE3) and SHuffle T7 Express were not discovered, but the VHH expressed by BL21 (DE3) maintained a degradation rate of 0.04. The degradation rate is described by the slope of time (h) versus the relative integrity.

In addition, Figure 4A shows that a polypeptide estimated its molecular mass by SDS-PAGE was 14 kDa, implying the digest production is generated by a trypsin cleavage after R20 instead of the next cleavage site R32 (digest production was 12.5 kDa). This might be caused by the incomplete formation of the first disulfide bond due to the oxidizing cytoplasmic environment, and R20 is close to the C23, for the disturbed C23–C97 disulfide bond would expose R20 to trypsin.

### 2.5. Thermal Stability

**Figure 5:**
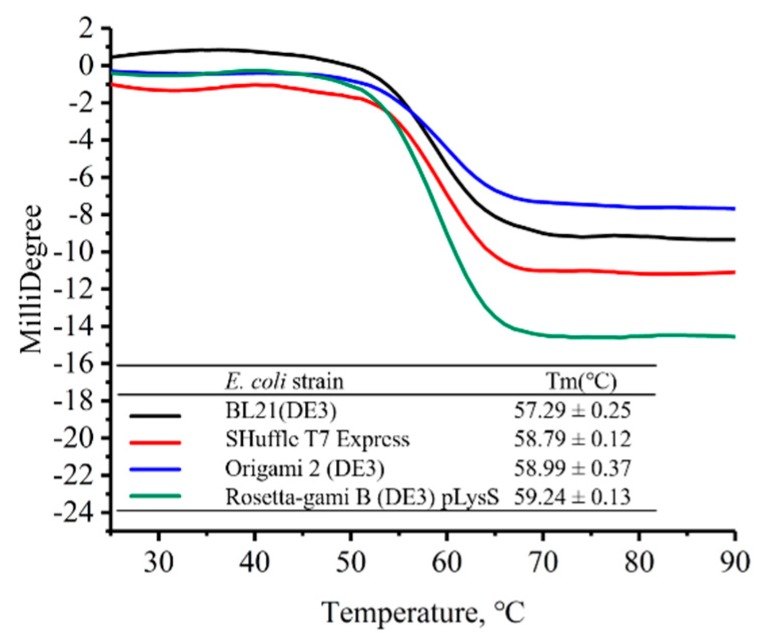
Thermal transition profiles obtained by molar ellipticity of anti-β2MG VHHs at 216 nm is plotted against temperature.

Herein, BioKine Analysis Software was used to analyze the far-UV CD spectral changes over a range of temperature from 25 to 90 °C at 1 °C steps in order to obtain the transition temperature (T_m_) of the VHHs. The far-UV CD spectrum of the VHH exhibited a negative feature peak at 216 nm due to the β-sheet structures of the VHH. As shown in Figure 5, a two-state transition process induced by heat denaturation was observed for all four VHHs. The table inset in Figure 5 shows the T_m_ values of the four VHHs, thus indicating that differences also exist in thermal stability when the soluble VHH expressed in different *E. coli* host cells. T_m_ of the VHH from Rosetta-gami B (DE3) pLysS was 59.24 ± 0.13 °C—slightly higher than the VHH expressed by SHuffle T7 Express (58.79 ± 0.12 °C) and the VHH expressed by Origami 2 (DE3) (58.99 ± 0.37 °C). The VHH from BL21 (DE3) had a less stable conformational state; its value of T_m_ was 57.29 ± 0.25 °C. The result here matches Figure 3, as the β-sheets bring more hydrogen bonds which make contributions to thermal stability.

### 2.6. Chemical Stability

**Figure 6:**
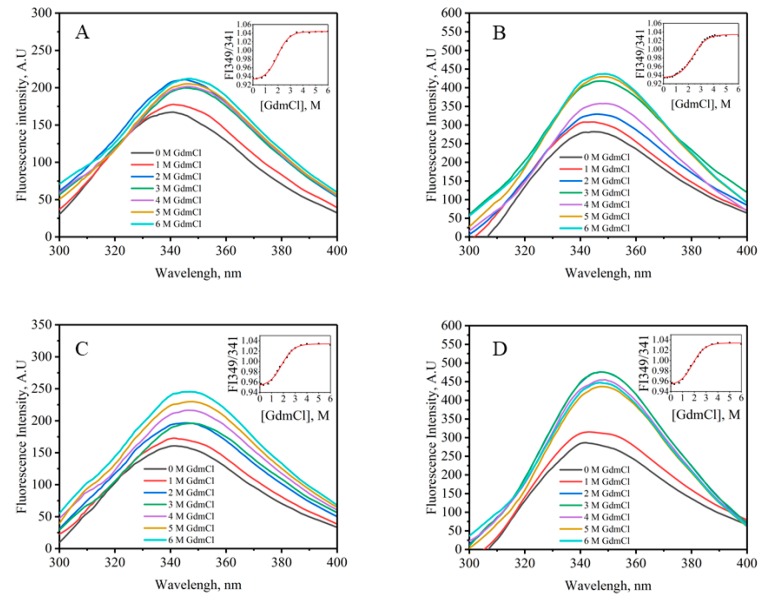
Fluorescence spectra of GdmCl-induced denaturation of VHHs expressed by BL21 (DE3) (**A**), SHuffle T7 Express (**B**), Origami 2 (DE3) (**C**) and Rosetta-gami B (DE3) pLysS (**D**). The insets were the denaturation curves obtained by plotting change in fluorescence intensity at 349 nm divided by intensity at 341 nm as a function of [GdmCl]. The protein concentration used was 0.2 mg/mL. Only a few spectra are shown for clarity.

Figure 6 displays GdmCl unfolding curves for VHHs, and the unfolding process was monitored by using the ratio of the fluorescence intensity at 349 and 341 nm. The data in Figure 6 suggest that the transitions are two-state [26], and the parameters were calculated by fitting the entire transition curves using Equation (1). Again, there was no significant difference between VHHs expressed in SHuffle T7 Express, Origami 2 (DE3) and Rosetta-gami B (DE3) pLysS according to Table 2, but the stability of the VHH expressed by BL21 (DE3) was inferior. The chemical stability further confirmed the less compact structure of VHHs produced in the BL21 (DE3), which suggests the incomplete formation of the disulfide bonds.

All results revealed there are dissimilarities in the soluble VHH productions in different *E. coli* hosts. For engineering our anti-β2MG VHH, Rosetta-gami B (DE3) pLysS was more suitable than the other three hosts: BL21 (DE3), SHuffle T7 Express and Origami 2 (DE3) serve as the host cell. It turns out that the expression environment is an important factor that should not escape our notice when choosing prokaryotic cytoplasmic expression.

## 3. Discussion

The simplified format of camelid VHHs avoids common bottlenecks in antibody generation and allows for a low-cost and less time-consuming manufacturing by expressing in the cytoplasm of *E. coli*. Two disulfide bonds exist in our anti-β2MG VHH, and this may be the reason for its robust structure [27]. However, the disulfide bonds in VHHs made it difficult to form soluble proper conformation in the reducing cytoplasm. Nevertheless, expression in the cytoplasm is still possible for the sake of engineered *E. coli* strains, like Origami (Novagen), Rosetta (Novagen) and SHuffle (NEB). The *E. coli* BL21 (DE3) strain has remained as the gold standard among prokaryotic expression hosts since the dawn of its commercialization [28]. BL21 (DE3) is deficient in the ion protease and lacks the ompT outer membrane protease that can degrade proteins during purification. Thus, the target proteins should be more stable. In this study, we obtained soluble form VHH in BL21 (DE3), and it showed a less stability than the other three, including thermal stability (Figure 5), trypsin resistance (Figure 4), and GdmCl tolerance (Figure 6).

The Origami 2 (DE3) strain has mutations in both the *trxB* and *gor* genes, which greatly enhance disulfide bond formation in the *E. coli* cytoplasm. SHuffle T7 Express has been recently prepared for expressing disulfide bonded proteins in its cytoplasm. In addition to mutations in the *trxB* and *gor* genes, a signal sequence disulfide bond isomerase *DsbC* gene is integrated into its chromosome [29]. The production of disulfide-bond containing recombinant proteins has been dramatically increased in the soluble fraction of cell lysates by using this strain. In this case, the VHHs expressed in Origami 2 (DE3) and SHuffle T7 Express demonstrated similar biological properties and were superior to the VHHs expressed in BL21 (DE3).

The Rosetta-gami B (DE3) pLysS strain has *trxB* and *gor* mutations. In addition, it supplies tRNAs for six rare codons on a compatible chloramphenicol-resistant plasmid. The anti-β2MG VHH expressed in Rosetta-gami B (DE3) pLysS exhibited the top properties of the four VHHs. The more oxidizing environment of Rosetta-gami B (DE3) pLysS contributes to disulfide bond formation to promote the proper folding of the VHH; therefore, the VHH has the highest affinity to β2MG and shows the highest stability to resist heat, trypsin, and chemical denaturant.

The canonical disulfide bond in nanobody folds is interior to the nanobody, and it is more resistant to reducing environments. However, the second disulfide bond is between CDRs, is usually more solvent exposed, and is therefore more susceptible to reducing environments. These findings for the anti-β2MG nanobody also apply to other nanobodies containing two or more disulfide bridges.

We emphasize that, although the periplasmic expression of disulfide-bonded VHHs would have been the preferred option, this approach is not devoid of drawbacks and often yields low expression levels due to limited space and an inefficient export of large proteins from the cytoplasm into the periplasmic compartment. As a consequence, we prefer the cytoplasmic expression and the pET expression vector for large-scale engineering. It might not be the optimal expression system for VHHs, and we will continue to optimize it in the future. For now, however, it is the more suitable option for our purposes in view of the fact that the considerable soluble yield of VHH in Rosetta-gami B (DE3) pLysS is approximately 55 mg per liter cultivated in the flask. It is worth noting that it is the same amino acid sequence, after all, so the differences are not very significant among the VHHs expressed in different hosts. However, these differences will be magnified by engineering and will become apparent. Therefore, it is necessary to explore the effect of the expression environment provided by the host on VHHs.

## 4. Materials and Methods

### 4.1. Expression of the Anti-β2MG VHHs

The anti-β2MG VHH gene sequence was generated in our lab, and the gene was subcloned into the pET23a expression vector with a 6×His purification tag. A linker (AHHSEDP), which comes from the camel IgG_3_ hinge region, was inserted between the VHHs and 6×His-tag [1]. The entire construction (VHH-AHHSEDP-6×His-tag) was named CNb1. After the sequencing of the CNb1 nucleotide sequence on the pET23a vector, the vector was transformed into BL21 (DE3), SHuffle T7 Express, Origami 2 (DE3), and Rosetta-gami B (DE3) pLysS for the VHH expression, respectively. *E.coli* cells that harbored the constructed vector were inoculated in 1000 mL of a TB medium supplemented with the corresponding antibiotics (specifically, BL21 (DE3); SHuffle T7 Express was inoculated into a medium with 100 μg/mL of ampicillin; Origami 2 (DE3) was inoculated into a medium with 100 μg/mL of ampicillin, 12.5 μg/mL of tetracycline and 50 μg/mL of streptomycin sulfate; and Rosetta-gami B (DE3) pLysS was inoculated into a medium with 100 μg/mL of ampicillin, 12.5 μg/mL of tetracycline, 34 μg/mL of chloramphenicol and 15 μg/mL of kanamycin) and were shaken at 200 rpm at 37 °C. Then, protein expression was induced at 200 rpm and 18 °C for 16 h with isopropyl β-d-1-thiogalactopyranoside (IPTG) with a final concentration of 0.25 mM. The bacterial cells were harvested, washed and resuspended with the equilibration buffer (10 mM PBS buffer, pH 7.4) at 4 °C.

### 4.2. Purification of the Recombinant Anti-β2MG VHHs

The cells were lysed by a high-pressure homogenizer (APV, NC, USA). Cell debris was removed by centrifugation at 15,000× *g* for 20 min. The supernatant was loaded onto a 5 mL HisTrap column (GE Life Sciences, MA, USA) using an AKTA FPLC system (GE Life Sciences, USA). Final purification was performed by size-exclusion chromatography (SEC) using a Superdex 75 (10/300) column (GE Life Sciences, USA) in a buffer (10 mM PBS, 0.2 mM PMSF, pH 7.4). The protein concentration was determined using the Bradford protein reagent with bovine serum albumin as the standard (Solarbio, Beijing, China). The processes of expression and purification of VHH were followed and analyzed using denaturing sodium dodecyl sulfate polyacrylamide gel electrophoresis (SDS-PAGE). Protein purity was quantitatively analyzed using Image Lab^TM^ software.

### 4.3. The Anti-β2MG VHHs Affinity Analysis

The affinities of the VHHs were determined by Biacore T200 (GE Life Sciences, USA) with Control software version 2.0.2 and Evaluation software version 3.1 was used for interaction analysis. The antigen β2MG was immobilized on a CM5 chip at pH 5.0 using the amine coupling kit (GE Life Sciences, USA). VHHs were injected at different concentrations (200 nM–1.56 nM, 2-fold serial dilution) into a running buffer (HBS-EP+, pH 7.4). The association phase was monitored for 200 s, and the dissociation phase was monitored for 300 s. The chip surface was regenerated after each cycle by injecting a 10 mM glycine-HCl buffer, pH 1.5 (30 μL/min, 45 s). The association rate constant *k_a_* and dissociation rate constant *k_d_* were calculated and analyzed using the monovalent analyte model, and the equilibrium dissociation constant (*K_D_*) was calculated (*K_D_* = *k_d_*/*k_a_*).

### 4.4. Circular Dichroism

Circular dichroism (CD) spectra were obtained using a spectropolarimeter (MOS 500, BioLogic, France) in a 10 mm path-length cell from 190 to 250 nm at room temperature with three scans averaged for each CD spectrum. The content of the protein secondary structure was calculated using the BioKine and Dicroprot software (BioLogic, France). Finally, total helix and sheets were obtained.

CD spectra and thermal denaturation measurements were performed on a spectropolarimeter with a Peltier temperature controller (BioLogic, France) using a 10 mm path-length quartz cuvette. Proteins were buffer-exchanged into a potassium phosphate buffer (10 mM potassium phosphate, pH 7.4) and prepared to a final concentration of 0.02 mg/mL. The thermal denaturation of VHHs was monitored by following the changes in ellipticity at 216 nm over a temperature range of 25–90 °C at a rate of 1 °C/min. The T_m_ of each VHH was determined using BioKine software (BioLogic, France). Each thermal melt experiment was performed in triplicate.

### 4.5. Trypsin Treatment

To investigate resistance of the VHHs to trypsin digestion, VHHs (1 mg/mL) were incubated at 37 °C with trypsin at a ratio of 100:1 (*w*/*w*) in 10 mM of PBS (pH 8.0) for 0.5, 4, 6 and 8 h. BSA was used as the control. The results of digestion were analyzed by SDS-PAGE on 15% separation gel and Image Lab^TM^ software. The resistance of the control group (sample without trypsin treatment) was considered as 100%.

### 4.6. Equilibrium Denaturation Experiments

GdmCl-induced unfolding followed by intrinsic fluorescence measurements was employed to determine the structural stability of the VHHs. The concentration of the denaturant stock solution was confirmed by measuring the refractive index [30]. Protein–GdmCl mixtures containing a final protein concentration of 0.2 mg/mL and denaturant concentrations ranging from 0 to 6.0 M GdmCl were prepared by adding the GdmCl stock solution (8 M GdmCl in 50 mM PBS, pH 7.4) to the purified protein (50 mM PBS, pH 7.4, 1 mM EDTA, 0.1 mM DTT). Samples were incubated for 24 h at 37 °C to attain equilibrium.

Fluorescence spectra were measured using Infinite M Nano (TECAN, Männedorf, Switzerland) at room temperature. The samples were excited at 270 nm, and the emission spectra recorded in the range of 300–400 nm. The transition curves of the intensity maxima at 341 nm served as a probe for the tertiary structure versus [GdmCl]. A non-linear least-squares method was used to analyze these curves [31]:

(1) $$y=y_{N}+y_{D}\times \mathrm{Exp}\left[-\left(∆G_{D}^{0}+m\left[d\right]\right)/RT\right]/\left(1+\mathrm{Exp}\left[-\left(∆G_{D}^{0}+m\left[d\right]\right)/RT\right]\right),$$

where *y* is the observed signal at FI 341, *y*_N_ and *y*_D_ are the properties of the native and denatured states of the protein under identical experimental conditions in which *y* has been measured, *R* is the gas constant, and *T* is the temperature in Kelvin. [d] is the molar concentration of denaturant. $∆G_{D}^{0}$ is the value of the Gibbs free energy change ($∆G_{D}$) in the absence of the denaturant. m is the slope ($\partial ∆G_{D}$/∂[denaturant]).

## 5. Conclusions

In this study, we studied the effect of different *E. coli* expression hosts on the properties of anti-β2MG VHHs. The result of SPR analysis demonstrated that the VHH expressed by Rosetta-gami B (DE3) pLysS has the highest affinity with a *K_D_* of 4.68 × 10^−8^ M. As measured by circular dichroism (CD) analysis, the structure of the VHH expressed by Rosetta-gami B (DE3) pLysS is the most stable and robust. And the VHH expressed by Rosetta-gami B (DE3) pLysS shows the highest stability to resist trypsin and GdmCl. Therefore, we give Rosetta-gami B (DE3) pLysS priority to engineer the anti-β2MG VHH.

## Figures and Tables

**Table 1:** Biacore analysis of the VHHs expressed by different hosts.

*E. coli* Strain	*ka* (10^5^ /Ms)	*kd* (10^−2^/s)	*K_D_* (nM)
BL21 (DE3)	1.21 ± 0.51	1.26 ± 0.72	100.09 ± 17.77
SHuffle T7 Express	3.02 ± 0.16	2.00 ± 0.78	69.83 ± 1.10
Origami 2 (DE3)	2.52 ± 0.12	1.65 ± 0.73	67.02 ± 4.79
Rosetta-gami B (DE3) pLysS	4.35 ± 0.70	2.00 ± 0.45	45.97± 6.01

**Table 2:** GdmCl-induced unfolding of VHHs expressed by different hosts ^a^.

*E. coli* Strain	Δ*G*_D_^0^, (kcal·mol^−1^)	*m*, (kcal·mol^−1^·M^−1^)	*C*_m_ (M)
BL21 (DE3)	2.90 ± 0.55	1.42 ± 0.18	2.25 ± 0.05
SHuffle T7 Express	5.61 ± 0.07	2.21 ± 0.10	2.54 ± 0.06
Origami 2 (DE3)	5.29 ± 0.17	2.20 ± 0.10	2.41 ± 0.04
Rosetta-gami B (DE3) pLysS	5.70 ± 0.05	2.22 ± 0.04	2.57 ± 0.02

^a^ Isothermal unfolding experiments were carried out in 50 mM PBS, 1 mM EDTA, 0.1 mM DTT, pH 7.4. A two-state unfolding model was fitted to the data.

## Supplementary Materials

The following are available online, Figure S1: Gel filtration chromatography profile of SEC; Figure S2: The SDS-PAGE pattern of VHHs after SEC; Figure S3: SPR sensorgrams of VHHs expressed in the four hosts.
