# Supplementary material for: High Expression Achievement of Active and Robust Anti-β2 microglobulin Nanobodies via E.coli Hosts Selection
Source: Molecules. 2019 Aug 7;24(16):2860. doi: 10.3390/molecules24162860 (PMC6720793; doi:10.3390/molecules24162860)
Supplement: Supplementary file 1 [file molecules-24-02860-s001.pdf]

Supplementary Materials

Sequence Information of Anti-β2MG VHHs (CNb1).

In the amino acid sequence, we substituted the corresponding amino acid residues in the CDR regions with X, because the related patent is in the process.

The nanobody is composed of 142 amino acids including the natively originated linker, and the molecular weight is 15812 Da. It shows the nanobody contains two pairs of disulfide-bound and the likely cleavage site R_{20} in the FR1 region is also exhibited.

AQVQLQESGGGSVQAGGSLRLSCAASXXXXXXXCXXWFRQAPGKEREWVARXXXXXX
XXYYADSVKGRFTFSQDNAKNTVYLQMDSLEPEDTATYYCXXXXXXXCXXXXXXXXXXXXX
WGQGTQVTVSSAHHSEDPHHHHHH

In the DNA sequence, codons in CDR regions were hidden by "-" and the rare codons shown in bold.

GCCCAGGTGCAGCTCCAGGAGTCTGGGGGAGGCTCGGTGCAGGCTGGGGGTCTCTG
AGGCTCTCCTGTGCCGCGTCT---------------CGT---TGC------TGGTTCCGCCAGGCCCCCGG
GAAGGAGCGTGAGTGGGTCGCACGT------------------------TACTATGCAGACTCCGTGAAG
GGCCGATTCACCTTTTCCCAGGACAACGCCAAAAACACGGTGTATCTGCAAATGGAC
AGCCTGGAACCTGAGGACACGGCCACTTATTACTGT---------------------TGC--------------------
-------------------TGGGGTCAGGGGACCCAGGTCACCGTGAGCTCAGCGCACCACAGCGA
AGACCCCCACCATCACCACCATCAT

Purification of Anti-β2MG VHHs (CNb1).

The VHHs were expressed as His-tag fusions and purified by HisTrap column (GE Life Sciences, USA) using an AKTA FPLC system (GE Life Sciences, USA). Final purification was performed by size-exclusion chromatography (SEC) using a Superdex 75 (10/300) column (GE Life Sciences, USA) in buffer (10 mM PBS, pH 7.4, 0.2 mM PMSF). The processes of expression and purification of VHHs were followed and analyzed using denaturing sodium dodecyl sulfate–polyacrylamide gel electrophoresis (SDS-PAGE). Protein purity was quantitatively analyzed using Image Lab ™ software.

Figure S1 and Figure S2 showed the elution fraction of 5–10 mL were impurities, and the elution fraction of 12.6–14.5 mL were purified VHHs and used in subsequent experiments. Compared with SHuffle T7 Express and Rosetta-gami B (DE3) pLysS, BL21 (DE3) and Origami 2 (DE3) had lower expression levels and more impure proteins.

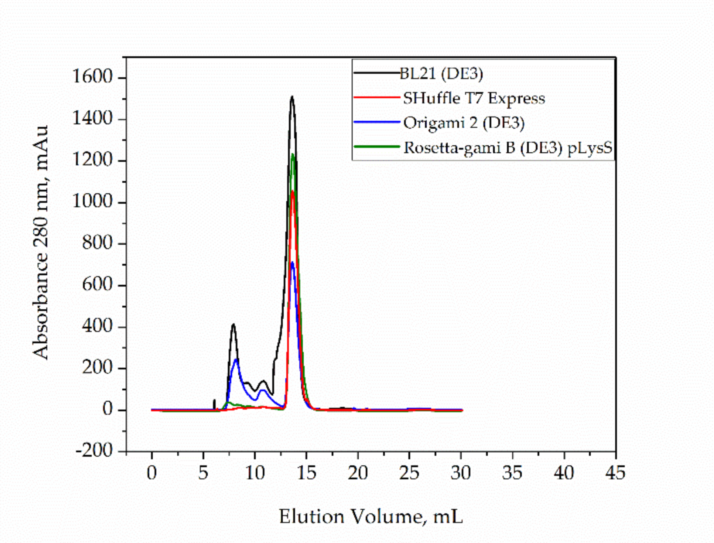

Figure S1. Gel filtration chromatography profile of the Ni-NTA purified VHHs, loaded onto a Superdex 75 column in PBS buffer.

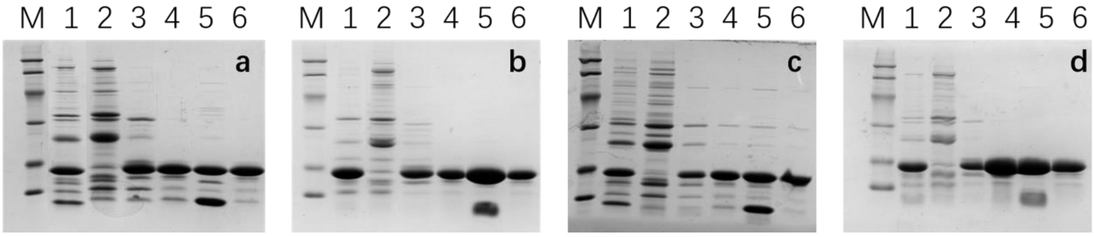

Figure S2. The SDS-PAGE pattern of VHHs after SEC. Lane 1, input sample (after Ni-NTA). Lane 2, elution fraction of 5–10 mL. Lane 3, elution fraction of 10–12.6 mL. Lane 4, elution fraction of 12.6–14.5 mL. Lane 5, elution fraction of 14.5–16 mL. Lane 6, purified VHHs. (a) BL21 (ED3). (b) SHuffle T7 Express. (c) Origami 2 (ED3). (d) Rosetta-gami B (ED3) pLysS.

Affinity Analysis of Anti-β2MG VHHs (CNb1).

The affinities of the VHHs were determined by Biacore T200 (GE Life Sciences, USA) with Control software version 2.0.2 and Evaluation software version 3.1 was used for interaction analysis. Figure S3 showed the multiple curves at different concentrations for each strain.

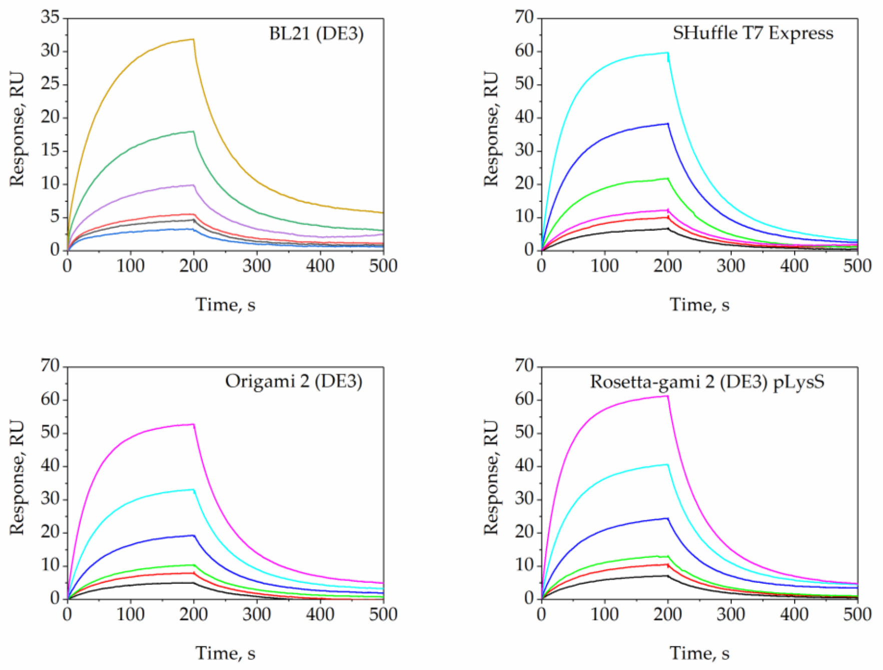

Figure S3. SPR sensorgrams of VHHs expressed in the four hosts. The range of VHH concentrations used in each experiment is from 50 nM to 1.56 nM. The kinetic and affinity constants are reported in Table 1.
